# A diode for ferroelectric domain-wall motion

**DOI:** 10.1038/ncomms8361

**Published:** 2015-06-10

**Authors:** J.R. Whyte, J.M. Gregg

**Affiliations:** 1 Centre for Nanostructured Media, School of Maths and Physics, Queen's University Belfast, University Road, Belfast BT7 1NN, UK

## Abstract

For over a decade, controlling domain-wall injection, motion and annihilation along nanowires has been the preserve of the nanomagnetics research community. Revolutionary technologies have resulted, like racetrack memory and domain-wall logic. Until recently, equivalent research in analogous ferroic materials did not seem important. However, with the discovery of sheet conduction, the control of domain walls in ferroelectrics has become vital for the future of what has been termed ‘domain-wall electronics'. Here we report the creation of a ferroelectric domain-wall diode, which allows a single direction of motion for all domain walls, irrespective of their polarity, under a series of alternating electric field pulses. The diode's sawtooth morphology is central to its function. Domain walls can move readily in the direction in which thickness increases gradually, but are prevented from moving in the other direction by the sudden thickness increase at the sawtooth edge.

## Abstract (web summary)

In ferromagnets, domain wall control is vital for applications like racetrack memory, whereas in ferroelectrics it could facilitate new electronics. Here, the authors demonstrate a diode for ferroelectric domain wall motion, where domain walls can move in one direction but not in the other.

## Graphical abstract

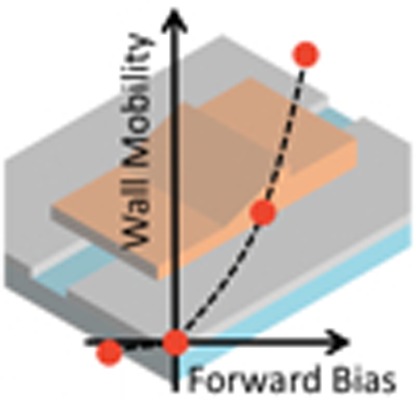

Over the last decade there has been an explosion of interest in low dimensionality conduction, such as that associated with graphene^1^, surface states of topological insulators^2^ and interfaces in LaAlO_3_-SrTiO_3_ bilayers^3^. Recent evidence that ferroelectric domain walls may constitute a completely new group of two-dimensional conductors^4^,^5^,^6^,^7^,^8^,^9^ is therefore genuinely exciting. This is reinforced by the realization that ferroelectric domain walls possess advantageous properties that established sheet conductors do not: they are mobile; can be controllably shunted from point to point; and can be spontaneously created or made to disappear. The genuine expectation is that conducting domain walls might be used in new ways, as dynamic functional entities, in new forms of devices (such as adaptive circuits) that push forward the embryonic field of domain-wall nanoelectronics^4^,^5^,^6^,^7^,^8^,^9^,^10^,^11^.

Meaningful progress, however, requires a much deeper understanding of the fundamentals of confined charge transport, including the role of defects^12^,^13^,^14^,^15^,^16^,^17^, and the development of a much greater level of control over individual ferroelectric domain-wall movement than has been obtained to date^18^,^19^, although recent progress in this respect by McGilly *et al*.^20^ is noteworthy. Established nanomagnetics research can help act as an inspiration: the controlled propagation of domain walls in thin strips of permalloy has already been used to make sophisticated demonstrator devices for both domain-wall logic^21^,^22^,^23^ and for racetrack memory^24^. In both of these technologies, strong emphasis has been placed on the effective development of a shift register capability, where the domain pattern along a permalloy nanowire can be moved in one direction without the overall growth or contraction of individual domains that would compromise the integrity of either the data storage or logic operation functions.

The key to developing a shift register is to find a way to move all domain walls in the same direction. This is not trivial. If a conventional field were applied to a set of domains under normal circumstances, those with dipoles oriented favourably with respect to the field would grow, while others would contract, such that domain walls of opposite polarity (defined by the specific sequence of adjacent domain orientations) would move in opposite directions (Fig. 1a). In Cowburn's domain-wall logic circuits^21^,^22^,^23^, a shift register was achieved by using a combination of carefully crafted wire geometries and a rotating H-field; in Parkin's racetrack memory^24^, it was achieved by passing a spin-polarized current along the nanowire such that the spin-torque momentum transferred to the domain walls, as a result of spin reorientation between domains, generated a common sense of domain-wall movement parallel to the net electron flow. Neither of these strategies can be readily adapted to generate ferroelectric shift registers: the discrete polarization directions in a ferroelectric unit cell mean that rotating electric fields would not lead to the same domain-wall dynamics as in nanomagnetics, and the physics associated with spin-polarized charge transport is not relevant to ferroelectrics.

An alternative shift register concept has been suggested and demonstrated by Franken *et al*.^25^ in permalloy nanowires: a potential profile consisting of a periodic series of sawtooth features was developed using inhomogeneous gallium implantation, introduced by differential local exposure to a focused ion beam (FIB). Domain walls, residing at local minima in this asymmetric potential landscape (Fig. 1b), experience a potential gradient in one direction of motion, which is significantly smaller than that in the opposite direction. A range of applied fields therefore exists, which will cause motion of domain walls of one polarity (up the relatively shallow potential gradient), but will be insufficient for domain walls of opposite polarity to move at all (the steep potential gradient at the sawtooth edge would be greater than the applied field). When the sense of the applied field is reversed, the domain walls that were previously mobile become pinned and the previously pinned walls are able to move. Importantly, the movement of the domain walls of opposite polarity under sequential alternating applied fields will all be in the same direction. As long as durations and magnitudes are optimized, each pair of opposite field pulses can result in the entire domain structure moving one wavelength along the sawtooth potential surface, hence generating the desired shift register effect (Fig. 1c).

Allwood *et al*.^26^,^27^ have shown that the critical sawtooth potential needed can also be realized morphologically, by patterning an arrowhead structure along an otherwise uniform ferromagnetic nanowire: in passing through the arrowhead, a domain wall, oriented perpendicular to the wire axis, must increase its area. In one direction this areal increase as a function of position is gradual, while in the other it is sudden. Since the total energy of the domain wall is proportional to its area (domain walls are characterized by a domain-wall energy per unit area^28^), the sawtooth morphology associated with the arrowhead is equivalent to a sawtooth potential landscape experienced by the domain wall. In this letter, we show that the same idea can be successfully used to make a diode for ferroelectric domain walls and that, hence, an analogous functioning ferroelectric shift register device is possible.

## Results

### Sample fabrication

**Figure 2:**
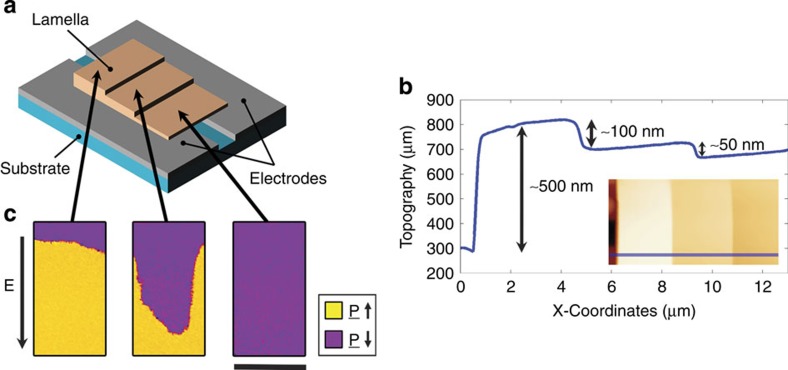
Experimental setup and domain dynamics of a terraced KTP lamella. Schematic illustration of a KTiOPO_4_ (KTP) ferroelectric lamella cut into terraces of different thickness and incorporated into a coplanar capacitor structure (**a**). In **b**, a line profile of the topography of a terrace-cut sample, measured using atomic force microscopy (AFM), is presented, showing well-defined steps in thickness. Inset is a plan-view image of the AFM topography scan and the trace of the line section shown is highlighted in blue. PFM scans (**c**) show the extent to which polarization reorientation occurs as a function of thickness from an initially fully poled state. In this terraced sample the thinnest region (∼400nm thick) has been fully switched in the reverse sense; in the region of intermediate thickness (∼500 nm) ∼60% switched and the thickest region (∼600 nm) only ∼15% switched. It appears that, in this geometry, thickness is strongly correlated with switching dynamics. The scale bar is 3 μm in length.

For initial investigations into the way in which thickness might affect domain-wall mobility, a terraced lamella (thin slice) was manufactured from a bulk single crystal of uniaxial ferroelectric KTiOPO_4_ (KTP) and integrated into a coplanar capacitor geometry (Fig. 2a). The terraced topography was FIB milled into one face of the lamella, while the other face was left flat to ensure good electrical contact with the coplanar electrodes. Atomic force microscopy (AFM) showed well-defined steps on the lamellar face (Fig. 2b). After completely poling this lamella in one sense, piezoresponse force microscopy (PFM) was used to image the extent to which switching occurred after a 1 s 60 V reverse pulse was applied across the capacitor. As is evident in Fig. 2c, the dynamics of switching were very different in the three regions of different thickness: switching was significantly accelerated in the thinnest section. This intriguing observation prompted a different strategy for site-specific domain-wall injection in the test diode structure subsequently fabricated than had been developed in previous research, where engineered local-field hot spots had been used^18^,^19^. Instead, here the newly established link between reduced thickness and a lowering in coercivity allowed a domain-wall injection pad, analogous to those used in nanomagnetism^29^,^30^, to be realized.

**Figure 3:**
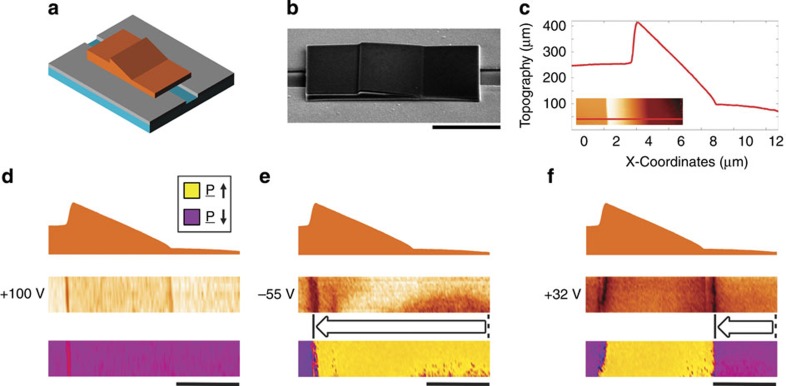
The ferroelectric domain-wall diode. Schematic illustration of a KTP lamella with a sawtooth cross-sectional morphology, straddling an interelectrode gap, in a coplanar capacitor device (**a**). The real device was imaged (**b**) using SEM (52° tilt); in **c**, a line profile of the lamellar surface topography along with a plan-view AFM image (inset), with the location at which the line profile was taken marked in red, are presented. The domain-wall injection and motion associated with switching this device are presented in **d**–**f**. In each case, the cross-sectional morphology (top) is aligned with the plan-view PFM amplitude (middle) and phase (bottom). The panels in **d** illustrate the initial fully poled monodomain state after the application of +100 V bias pulse. In **e**, a partially switched state (after the application of −55 V) is given: a new domain wall has been injected and progressed from right to left up the ramp and over the sawtooth edge. In **f**, the state after a further bias pulse (+32 V) was applied in the original poling sense is presented: a domain wall of the opposite polarity has been injected into the device and moved to the base of the ramp. Little movement in the original wall injected in **e** has occurred, due to the potential barrier associated with the steep ledge of the sawtooth structure. The scale bar in **b** is 5μm long, while those in **d**–**f** are 3μm long.

The ferroelectric diode structure was therefore designed with a relatively thin parallel-sided region, to encourage domain-wall injection, adjacent to a section of gradual thickness increase up to a maximum point, followed by a sudden drop to another parallel-sided region of intermediate thickness (in which it was hoped domain-wall injection would be suppressed in comparison to the thin region); a schematic is shown in Fig. 3a. The resultant cross-sectional sawtooth morphology of the lamella (equivalent to a sawtooth domain-wall potential profile) can be seen in Fig. 3b,c showing a scanning electron microscopy image and AFM topography profile, respectively, of the integrated capacitor structure.

### Diode Behaviour

Figure 3d–f summarize the switching behaviour observed: initially, the entire KTP structure was poled to a monodomain state using a +100 V setting pulse; the sense of the applied field was then reversed, but deliberately set to a lower magnitude (−55 V) to avoid complete switching to an oppositely oriented monodomain state; this allowed the mechanics of switching to be observed through the formation of partially switched states. A reverse domain nucleated in the thinnest region of the diode structure, as expected from the discussion above. While one domain wall was completely expelled out of the sidewall of the domain-wall injection pad (as occurs in domain-wall injection pads used in nanomagnetics), the other wall propagated up the ramp and over the sawtooth edge, from right to left in Fig. 3e. When the sense of the field was changed back to that of the original poling pulse, at a further reduced bias level (+32 V), a new domain was again injected into the thinnest region of the structure. As before, one of the domain walls was expelled, while the other again progressed from right to left. If larger poling fields or longer pulse durations were used in the positive sense, domain-wall annihilation occurred and a monodomain, fully switched, state resulted (the starting state for the switching sequence described was recovered). There are several important aspects to the switching sequence that should be highlighted: first, that the domain injection pad of lower coercivity appears to work well and in an analogous manner to that found in nanomagnetics research^29^,^30^, allowing domain walls of opposite polarity to be successively and controllably injected into the active device region, while other domain walls are expelled; second, that the steep ledge of the sawtooth potential was extremely effective in preventing domain-wall movement in the device, such that, irrespective of the domain-wall polarity, all domain-wall movement occurred in the same sense (right to left).

**Figure 4:**
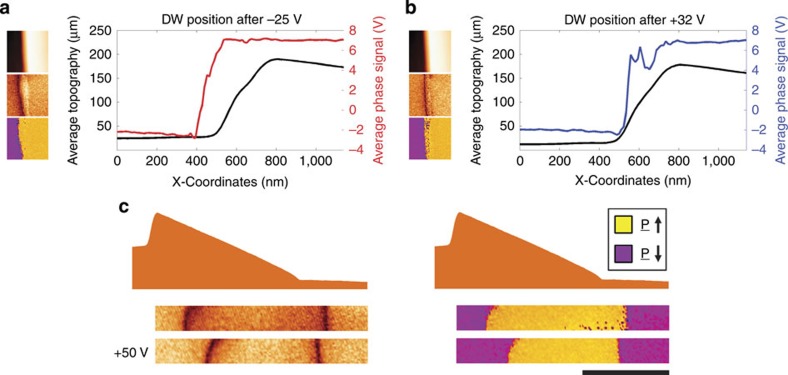
Domain pinning at ledge and increased mobility on moving down the ramp. (**a**,**b**) PFM scans on the left showing the topography (top), amplitude (middle) and phase (bottom) with the average phase and topography signals displayed in a graph on the right, highlighting the domain-wall position in relation to the ledge of the ramp. (**a**) −25 V moved the domain wall off the ledge from the position shown in Fig. 3e. (**b**) +32 V moved the domain wall back to the ledge where it remained pinned. (**c**) PFM information of the morphology (top), amplitude (left) and phase (right), of two domain walls where one is situated in the ramp and the other in the injection pad. After the application of a +50 V pulse, the domain wall in the ramp moves further than that in the injection pad, highlighting the domain-wall preference to move into regions of lower thickness (and hence lower potential). Scale bar is 2 μm in length.

Figure 4a,b highlights the effectiveness of the steep ramp ledge in preventing domain-wall motion: a domain-wall which was initially driven up the shallow ramp slope and just over the edge of the ramp using a negative applied voltage (−55 V as above) was moved further from right to left using a −25 V pulse. By then reversing the pulse, to the +32 V value used to generate the switching seen in Fig. 3f, the domain wall is seen to nestle into the steep edge of the ramp, but not progress further. The potential barrier associated with the sharp edge of the ramp is obviously key to the realization of the ferroelectric domain-wall diode demonstrated. In Fig. 4c a domain wall has been moved up the shallow incline of the ramp, but has not been moved over the ledge (it stopped short of the ledge during the initial switching pulse). On reversal of the field, this domain wall moves readily down the ramp, as it is not pinned. Indeed, its motion is enhanced relative to another wall travelling in a flat part of the diode structure: its wall area is decreasing as it moves and it is thus travelling down the potential gradient.

It should be noted that throughout the study described, it has been assumed that the domain walls examined are all flat planar objects and that their position mapped at the top surface of the KTP is representative of that through the entire thickness of the ferroelectric; while this assumed planar geometry should minimize the domain wall area and hence energy, which has been shown to be central to the successful operation of the wedge diode, some positional meandering through thickness could, in principle, occur. Indeed, finite element modelling shows that minor local variations in field distribution are present as a function of thickness, so that domain walls at the bottom surface of the KTP experience a slightly different environment from those at the top. In particular, in the thicker regions of the wedge structure, small hot spots at the electrode–ferroelectric interface begin to develop, which are not obvious in thinner regions. These hot spots might be thought to be likely points for domain-wall injection as demonstrated in previous research^18^. However, their influence appears to be swamped by the marked increase in switching dynamics seen in the thinner sections of the wedge device, where hot spots are not apparent. Nevertheless, through-thickness imaging, as performed recently by Kämpfe *et al*.^31^, could be useful for a more complete characterization of the domain-wall diode behaviour.

In summary, the notion that the total energy of a domain wall is proportional to its area has been used to design a sawtooth energy landscape through machining a ferroelectric lamella with a sawtooth thickness profile. This lamella has been integrated into a capacitor structure and the domain dynamics resulting from pulsed electric fields monitored using PFM. Distinct asymmetry in the domain-wall mobility has been demonstrated: domain walls can progress readily up the shallow incline of the sawtooth, but are prevented from moving in the opposite direction by the steep potential gradient at the sawtooth ledge. Effectively, a domain-wall diode has been demonstrated; by extension, in principle, the possibility of a shift register structure for ferroelectrics, analogous to those critical for domain-wall logic and racetrack memory applications in nanomagnetics, has also been shown. The fabrication methodology presented is clearly only relevant for a proof-of-principle device. However, the fact that Franken *et al*.^25^ were able to develop wedge-shaped variations in coercivity in a planar ferromagnetic thin film, by differential ion-beam exposure, gives us hope that similar techniques might be transferable to planar ferroelectric thin films; this could open the door for the creation of ferroelectric domain-wall diodes and shift registers using more conventional fabrication techniques.

## Methods

### Sample preparation

All capacitor structures investigated were fabricated by cutting lamellae from a bulk single crystal of KTP, using a FEI200TEM FIB, and placing them across a FIB-milled interelectrode gap (2 μm in width) on platinum-coated Al_2_O_3_ substrates. Thermal annealing (300 °C for 6 h) was used to partially recrystallize the FIB-induced damage at the surface of the lamellae and expel gallium in the form of oxide platelets, which was removed via 3 min of acid etching in a 3 M HCl solution. Localized electron beam deposition of platinum at lamella edges (using a FEI Nova 600 DualBeam system with a MeCpPtMe_3_ precursor gas) ensured good electrical contact and mechanical robustness of the completed coplanar capacitor device. Any deliberate local variations in lamellar thickness were developed on the top surface only, as a flat lower surface was needed to ensure sound electrical contact. Finally, each electrode of the sample was conductively secured with silver paste onto a glass slide with macroscopically patterned sputter-coated platinum electrodes, enabling application of voltage across the sample, whilst under an AFM scanner head.

### PFM domain imaging

PFM measurements were carried out using a Veeco Dimension 3100 AFM system with a Nanoscope IIIa controller, modified for PFM measurements using a EG&G 7265 lock-in-amplifier. Here an oscillating voltage of 2 V_RMS_ at a frequency of 20 kHz was applied to a Nanosensors PPP-EFM cantilever (force constant ∼2.8 N m^−1^). The d.c. voltages were applied to lamellae using a Keithley 237 High Voltage Source Measure Unit.

## Additional information

**How to cite this article:** Whyte, J. R. & Gregg, J. M. A diode for ferroelectric domain-wall motion. *Nat. Commun.* 6:7361 doi: 10.1038/ncomms8361 (2015).

## Figures and Tables

**Figure 1:**
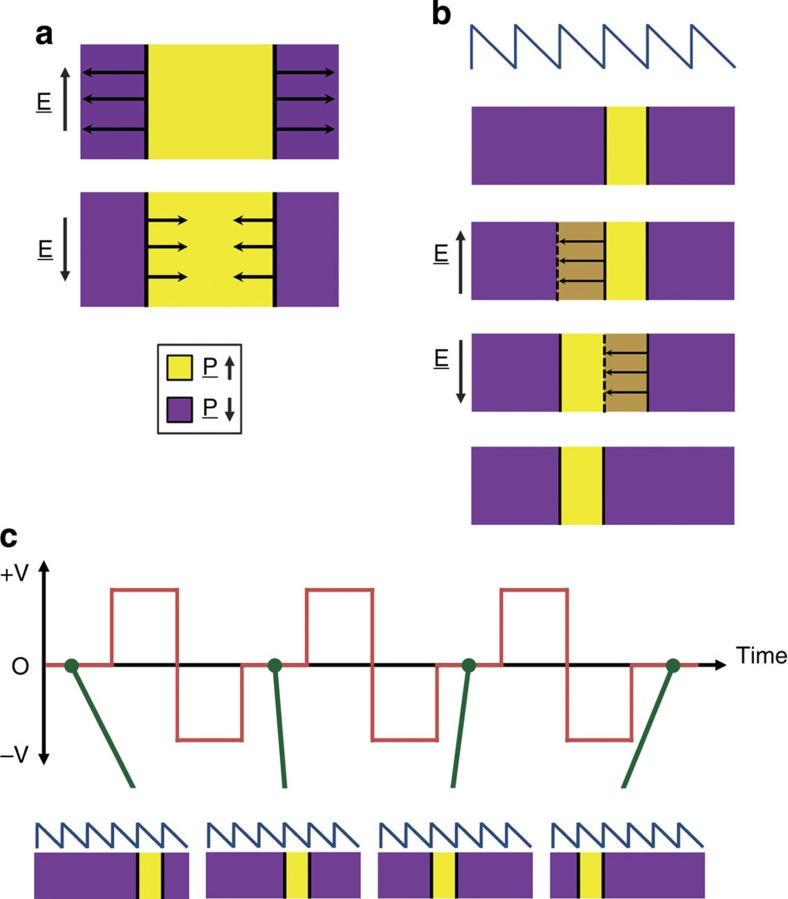
The working principle of a ferroelectric shift register. Under conventional circumstances, applied field pulses cause domain walls of opposite polarity to move in opposite directions, as domains in which polarization is favorably aligned with the applied field expand, while others contract (**a**). However, if the potential landscape can be designed as a series of saw-teeth, then, for a range of applied field magnitudes, domain walls of one polarity are frozen into position while those of opposite polarity move (**b**). If successive alternating field pulses are used of the right magnitude and duration, then domain walls of different polarity can move alternately all in the same direction and a domain-wall shift register based on ratchet-like domain-wall motion can be realized (**c**). The key building block is the diode responsible for the repeating unit in the sawtooth potential.
